# Leakage-Aware Machine Learning and Deep Learning Benchmarking of Food Antioxidant Capacity Prediction on the Antioxidant Food Table

**DOI:** 10.3390/antiox15091157

**Published:** 2026-09-11

**Authors:** Erkan Caner Ozkat

**Affiliations:** Department of Mechanical Engineering, Faculty of Engineering and Architecture, Recep Tayyip Erdogan University, 53100 Rize, Türkiye; erkancaner.ozkat@erdogan.edu.tr

**Keywords:** antioxidant capacity, machine learning, deep learning, data leakage, food quality

## Abstract

The Antioxidant Food Table is the largest open collection of measured food antioxidant capacity. It covers 3139 products assayed by the ferric reducing ability of plasma (FRAP) method. The table has served mainly as a dietary lookup source and has never been machine-readable or benchmarked. Here it was extracted into a validated open dataset (3135 records, 99.9%). A leakage-aware benchmark of antioxidant capacity prediction from product description and category was then constructed. Eighteen predictors, from naïve baselines to deep networks and fusions, were evaluated under two partitioning regimes with the same five seeds and permutation controls. Since 39.4% of records share a product name, the conventional random split rewards memorization. A learning-free duplicate lookup explained 45% of the apparent *k*-nearest-neighbor advantage over the category median. In grouped evaluation, ridge regression on term frequency–inverse document frequency (TF–IDF) features ($R^{2}=0.674$) outperformed both deep networks. Pretrained word vectors did not close this gap. An equal-weight fusion of all eight models performed best ($R^{2}=0.684$) with 2.6-fold lower variability. Protocol and representation, rather than architecture, dominated the outcome on this benchmark. The dataset, code, and predictions are released openly.

## 1. Introduction

Antioxidant capacity has become a central quality descriptor along the modern food supply chain. It informs raw material selection, processing decisions, shelf-life management, and consumer-facing claims [1,2,3]. As compositional information grows in volume and heterogeneity, the food sector has turned to artificial intelligence and other bioinformatic technologies. Applications of machine learning (ML) to antioxidant-related problems have multiplied in recent years [4,5,6]. Examples span the singlet-oxygen-scavenging activity of pure compounds [7] and the radical-scavenging capacity of doped nanoparticles [8]. In silico antioxidant assessment has also been advocated for in drug design [9]. Phenolic constituents have been predicted from mid-infrared spectra [10] and from cultivar descriptors via artificial neural networks [11]. Untargeted metabolomics has been coupled with ML or neurofuzzy logic to model phenolic biosynthesis [12,13]. Chemometric methods have characterized and authenticated olive oils, wines, cereals, hemp, and honeys from their phenolic fingerprints [14,15,16,17,18]. A single metal-oxide gas sensor has graded tea quality through classical ML and deep learning (DL) [19]. ML has also optimized in vitro regeneration protocols in cotton [20]. Furthermore, ML has been used to mine food–health associations from dietary and metabolomic data [21,22,23], and DL has supported antioxidative peptide discovery [6]. Beyond the food domain, hybrid analytical-to-AI modeling has matured for pulsating non-Newtonian heat flow [24], complementing physics-based force models of maglev transport [25].

Open food composition databases underpin much of this activity as shared infrastructure [26]. A prominent example is Phenol-Explorer [27,28]. It serves as a suspect-list source for phenolic screening [29]. The database also supplies a compound library for the virtual screening of dietary polyphenols against protein targets [30,31]. Related in silico campaigns have designed flavonoid-, terpenoid-, and chalcone-based bioactive compounds [32,33,34]. Phenol-Explorer further feeds metabolism-prediction tools [35] and the design of dietary interventions [36]. These uses treat the databases as reference libraries recording which compounds exist in foods. Their quantitative content values are rarely exploited as predictive features. Moreover, measured antioxidant capacity has not previously been the target of a systematic prediction benchmark.

Progress of this kind depends on open, well-characterized data. For food antioxidant capacity, the largest resource remains the Antioxidant Food Table of Carlsen et al. [37]. It reports 3139 foods, beverages, spices, herbs, and supplements measured with a modified ferric reducing ability of plasma (FRAP) assay [38]. In the open-access literature, however, it has mainly served as a lookup table. Epidemiological studies match food frequency questionnaire items to the tabulated values, sum them into a dietary total antioxidant capacity (TAC) score, and relate it to health outcomes. Dietary TAC derived in this way has been associated with cardiovascular risk profiles [39] and with type 2 diabetes, prediabetes, and insulin resistance [40,41,42]. Further reported associations concern obesity and body composition [43,44], ovarian and prostate cancer [45,46], and mortality and oxidative balance [47]. Other studies address cirrhosis severity [48], kidney function [49], semen quality [50], and diet quality in childhood cancer survivors [51]. Related work also covers nutritional status in water immersion [52], dietary adequacy across meal patterns [53], and nutritional resilience [54]. The matching step is explicitly manual. Li et al. [42], for instance, state that “the antioxidant value of the nearest comparable food was assigned”. Despite more than a decade of such use, the table has never been machine-readable or, to the author’s knowledge, served as a predictive benchmark.

The nearest attempt at the task shows why such a benchmark needs care. Guardado Yordi et al. [4] predicted the oxygen radical absorbance capacity (ORAC) of foods from flavonoid composition data. They reported a coefficient of determination of 0.957 for a random forest. In their data layout, however, each food contributes multiple rows, one per flavonoid, while the prediction target is defined at the food level. The training and test partitions were drawn at random over rows. Consequently, the same food appears on both sides of the boundary, and the reported accuracy largely reflects memorization. This failure mode, data leakage, is a leading cause of over-optimistic results across ML-based science [55]. Blocked or grouped cross-validation has long been recommended for structured data [56]. Within food chemistry, Hussein et al. [57] examined rooibos infusions and showed that published ML successes can be artifacts of duplicate measurements straddling the split. Once the evaluation was repaired, ML did not outperform a simple statistical baseline. The author’s electronic-nose benchmarking reached a similar conclusion for meat freshness [58]. In a cherry-orchard study, models tuned on a random within-season split saw their error nearly double in the next season [59]. Adjacent antioxidant ML applications likewise rely on random row-level splits [7,8]. Nevertheless, leakage-aware protocols remain rare in the food antioxidant ML literature.

Therefore, this study constructed the missing benchmark and quantifies the influence of evaluation protocol on apparent model performance. The Antioxidant Food Table was first converted into a validated, machine-readable dataset. Capacity was then predicted from the two inputs the table provides: the free-text product description and the food category. This regression task is distinct from food-entry matching, a retrieval problem revisited in the Discussion. Two regimes were evaluated in parallel: the random split that dominates the literature, and a grouped split in which no product name may straddle the boundary. The comparison spans naïve baselines, classical ML, character-level DL, and integration strategies, instrumented with permutation controls and a memorization baseline isolating duplicate lookup. Antioxidant capacity is treated throughout as a compositional quality indicator, not a predictor of physiological effect. This stance follows criticism of in vitro assays as health proxies [1].

The main contributions of this study are fourfold:

(i) The first machine-readable release of the Antioxidant Food Table, validated against the source publication;
(ii) The first leakage-aware benchmark of food antioxidant capacity prediction, comprising eighteen predictors under two partitioning regimes with permutation controls;
(iii) A quantitative decomposition attributing 45% of the apparent nearest-neighbor advantage over the category median to duplicate-name lookup;
(iv) An assessment of ML–DL integration showing that prediction-level fusion, rather than representation injection, yields the best observed grouped-regime performance.

The remainder of the paper is structured as follows: Section 2 describes the dataset construction, split design, model families, and evaluation protocol. Section 3 presents the results. Section 4 discusses the findings and their limitations, and Section 5 concludes the study.

## 2. Materials and Methods

**Figure 1:**
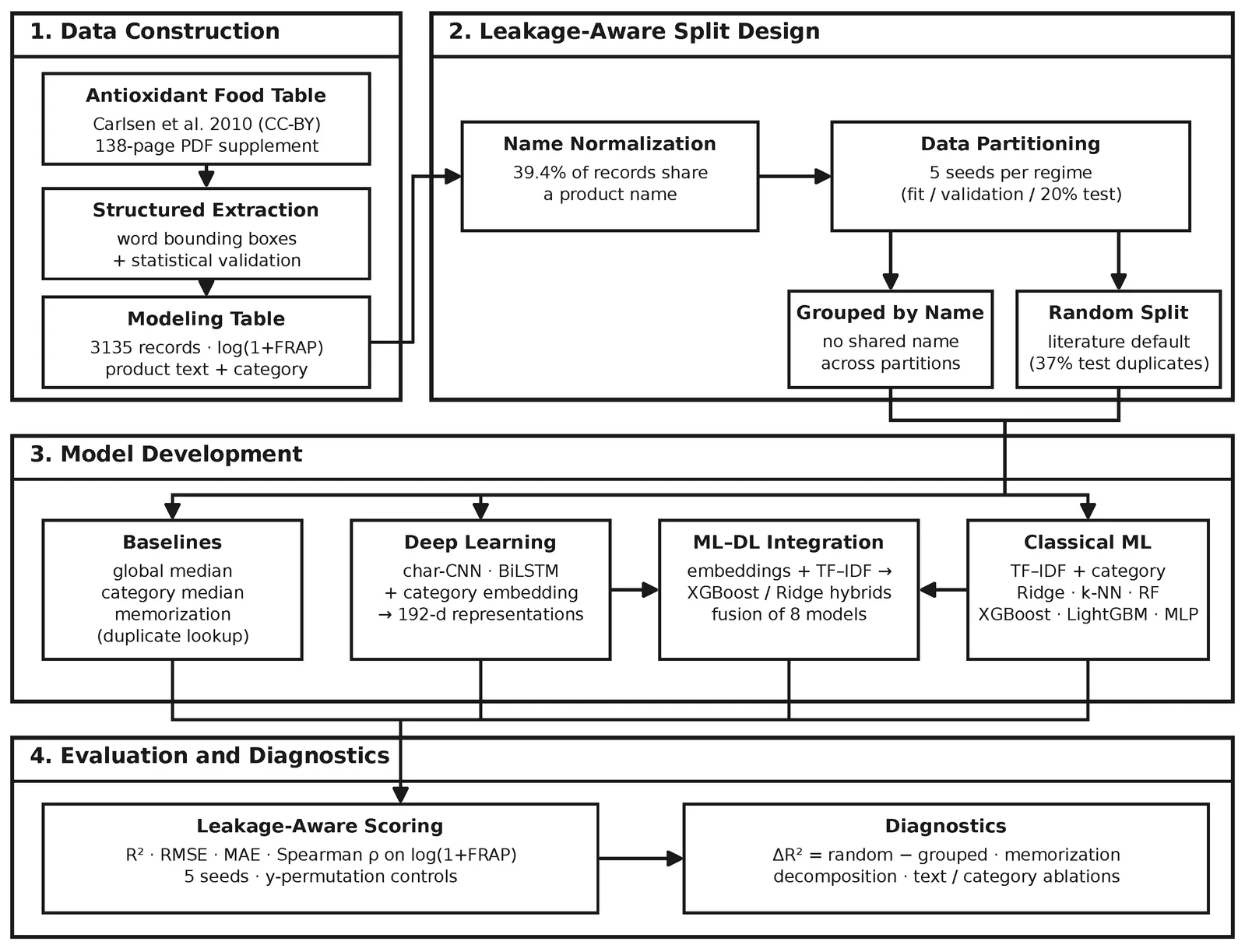
The flowchart of the proposed methodology. The Antioxidant Food Table was compiled by Carlsen et al. [37].

The methodology involved four main steps, as illustrated in Figure 1: 1. Data Construction, 2. Leakage-Aware Split Design, 3. Model Development, and 4. Evaluation and Diagnostics. All computations were performed in Python 3.12.3 (Python Software Foundation, Wilmington, DE, USA). The libraries were scikit-learn 1.2.2 [60], XGBoost 3.2.0 [61], LightGBM 4.6.0 (Microsoft Corporation, Redmond, WA, USA) [62], and PyTorch 2.6.0 (PyTorch Foundation, San Francisco, CA, USA) [63]. Both scikit-learn and XGBoost are community-maintained open-source libraries. All experiments were executed on the central processing unit (CPU) of a single workstation (Intel Core i7-7700HQ, 32 GB RAM; Intel Corporation, Santa Clara, CA, USA). Exact package versions, frozen partitions, per-run predictions, and trained-model outputs are provided in the accompanying repository, so every reported number can be reproduced.

### 2.1. Data Construction

The source material is the Antioxidant Food Table provided by Carlsen et al. [37] as a 138-page supplementary document. It tabulates 3139 products with category, description, manufacturer or origin, procurement country, and FRAP antioxidant content in mmol/100 g [38]. The table’s Creative Commons Attribution (CC-BY) license permits redistribution of derived data with attribution. Plain-text extraction proved unreliable because the layout shifts between pages and long names wrap across lines. Instead, word-level bounding boxes were obtained for every page. Each antioxidant value anchored a row band, and words were assigned to rows and columns by their coordinates. This procedure recovered 3135 of the 3139 records (99.9%) across all 24 categories.

**Figure 2:**
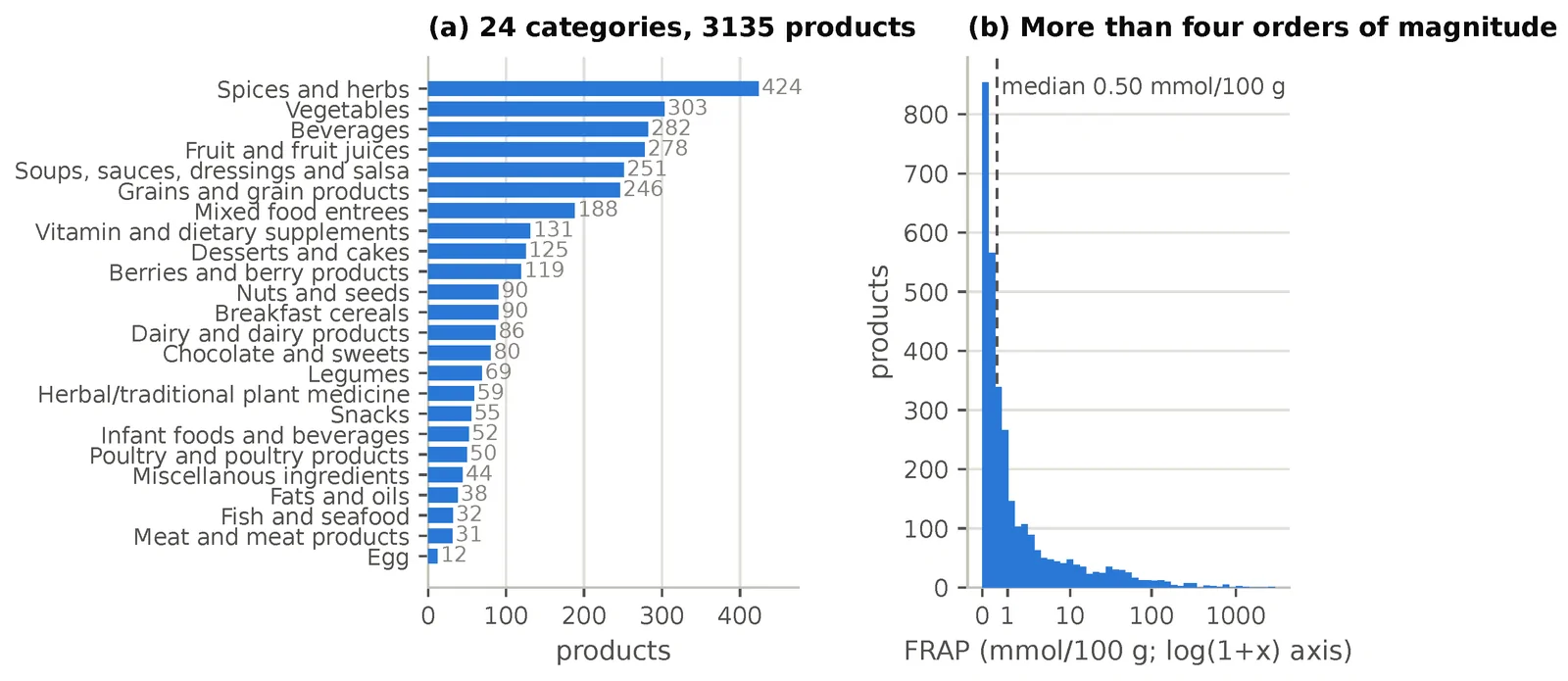
The extracted dataset: (**a**) records per category; (**b**) distribution of the FRAP content on a log(1+x) axis.

The extraction was validated in two stages. First, an independent value-level census of the supplement was compared with the extracted records. Per-category record counts and value sums match exactly in 23 of 24 categories. The single difference is one unrecovered record of 0.17 mmol/100 g among the mixed food entrees. Second, recomputed per-category statistics agree with the article’s summary table within published rounding for most categories, and every residual difference traces to a source-internal inconsistency. Nineteen products are printed under *Grains and grain products* in the supplement but counted under *Snacks* and *Desserts and cakes* in the article. Three records tallied in the article are absent from the supplement, and two summary values disagree with the supplement’s own listings. The supplement’s placement was preserved, and every inconsistency is documented in the released dataset. The category name *Miscellanous ingredients* retains the source’s verbatim spelling. The resulting modeling table contains the product description, the category, and the target variable for each record (Figure 2). Antioxidant content spans more than four orders of magnitude, from 0 to 2897.11 mmol/100 g, with a median of 0.50 mmol/100 g. The target was therefore modeled as log(1+FRAP), which accommodates the 40 exact zeros. These zeros concentrate in bottled waters, soft drinks, and refined ingredients such as salt, sugar, corn syrup, and distilled vinegar. A tabulated zero indicates a value below the two-decimal reporting resolution of the source, not a certified absence of antioxidant capacity. The FRAP assay also misses glutathione and other low-molecular-weight thiols, so the zeros are assay-relative [37,38].

### 2.2. Leakage-Aware Split Design

**Figure 3:**
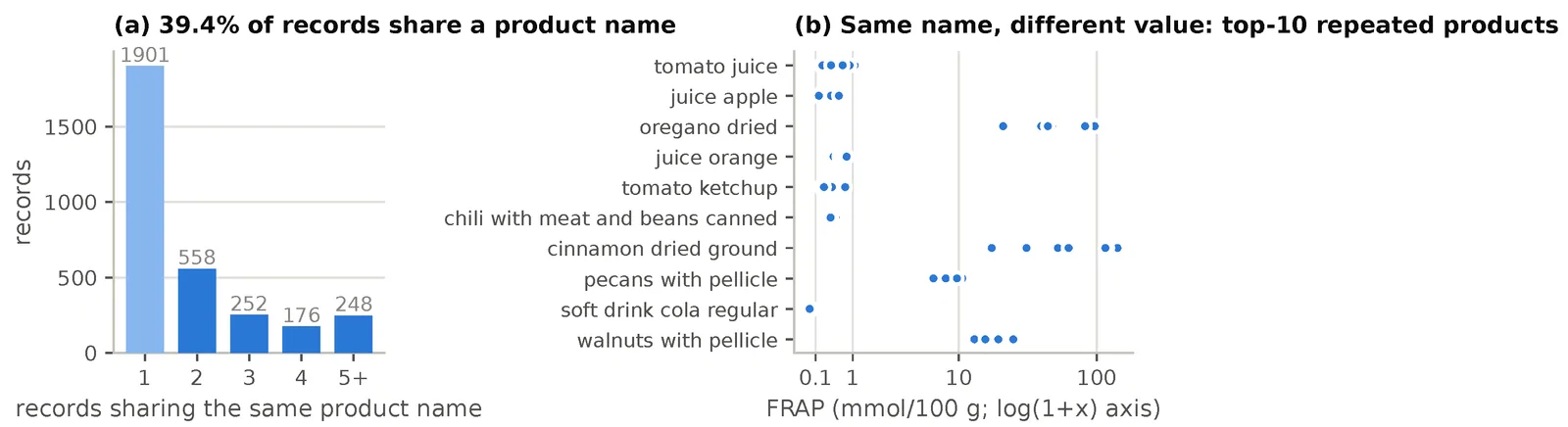
The leakage surface: (**a**) records by name multiplicity; (**b**) FRAP values of the ten most repeated product names.

Product names were normalized by Unicode decomposition, lower-casing, removal of non-alphanumeric characters, and whitespace collapsing. With this normalization, 39.4% of all records (1234 of 3135) share their product name with at least one other record (Figure 3a). These duplicates are not errors; they are the same food procured as a different brand, lot, or origin. For example, *tomato juice* appears eleven times and *oregano, dried* nine times, spanning 21.4 to 96.6 mmol/100 g (Figure 3b). A duplicated name therefore informs, but does not determine, the target value.

Two partitioning regimes, five seeds each, were frozen before any model was trained. In the random regime, records were assigned uniformly at random, the prevailing practice in the literature [4]. In the grouped regime, partitioning was performed over normalized product names [56]. No name may occur on both sides of any boundary. Each split reserves 20% for testing; the remainder divides into a fit partition and a validation partition (15% of the pool). The validation partition, group-aware in the grouped regime, is used only for early stopping and hyperparameter selection. Under the random regime, 34.6–38.0% of test records (mean 36.5%) share a product name with a training record. The grouped regime contains no such overlap by construction. The grouped regime is described as duplicate-controlled rather than leakage-free, since it eliminates the identified duplicate-name leakage but cannot exclude every conceivable source. Model comparisons are paired within a regime: for a given regime and seed, every model is scored on the identical test partition. Cross-regime contrasts use the same seeds but different test partitions. The two regimes nevertheless sample statistically similar test sets. Across seeds, the Kolmogorov–Smirnov statistic between the grouped and random test-target distributions averages 0.050, and no seed rejects equality (all p≥0.10). Mean test targets differ by at most 0.09 log units, and the largest category-share difference in any seed is 4.5 percentage points. Composition differences of this size cannot explain the regime gaps reported below, which concentrate in memorizing models and vanish for the representation-matched multilayer perceptron (MLP).

### 2.3. Model Development

Eighteen predictors were developed and organized into four families. The first family provides learning-free baselines, and the second comprises classical ML models on engineered features. The third family contains character-level DL networks, and the fourth integrates the classical and deep approaches. Each family isolates one question about the sources of predictive performance.

The first family establishes three learning-free reference points. The global median and the per-category median of the training pool provide performance floors. The third reference is a memorization baseline. It assigns each test product the mean measured value of identically named training products. Where no duplicate exists, it falls back to the training category median. Under the grouped regime, the memorization baseline finds no duplicates and must reproduce the category median exactly. This equivalence is a built-in correctness check, and the implementation satisfies it bit-identically. Under the random regime, the baseline quantifies the performance obtainable by duplicate lookup alone.

The second family applies six classical ML models to engineered features. Product descriptions were represented by term frequency–inverse document frequency (TF–IDF) features over word unigrams and bigrams (10,000 features) and character 3–5-grams (20,000 features). A one-hot category encoding was concatenated, and all vocabularies were fitted on the fit partition only. Ridge regression selected its regularization strength on the validation split from {0.1,0.3,1,3,10,30}. The *k*-nearest neighbors (*k*-NN) model used cosine distance, with *k* selected on the validation split from {1,3,5,10}. The random forest used 300 trees, $\sqrt{p}$ features per split, and a minimum leaf size of two, fixed a priori. A post hoc sensitivity grid over the latter two choices is reported in Section 3.1. XGBoost and LightGBM used a learning rate of 0.05, with the tree count set by early stopping on the validation split. XGBoost used a fixed depth of six levels with row and column subsampling of 0.8. LightGBM used a fixed budget of 63 leaves and column subsampling of 0.8, without a depth limit. An MLP with one hidden layer of 256 units was trained with per-epoch validation-based early stopping. The *k*-NN model is the direct automation of the manual “nearest comparable food” assignment used in the dietary literature [42]. The MLP is a representation-matched counterpart to the deep networks: the same optimizer family but engineered features.

The third family contains two character-level DL architectures implemented in PyTorch [63]. The first is a convolutional neural network (CNN) with parallel kernels of widths 2–5 over learned character embeddings [64]. The second is a bidirectional long short-term memory (BiLSTM) network [65] with masked max-pooling over time. Both receive a 16-dimensional category embedding and produce a 192-dimensional penultimate representation. Both were trained with Adam with validation early stopping. The character vocabulary was built from the fit partition only.

The fourth family integrates the classical and deep approaches at two levels. At the representation level, three hybrid predictors were formed. The first trains XGBoost on the concatenated 384-dimensional CNN and BiLSTM representations alone. The second and third train XGBoost and ridge regression on the TF–IDF and category features augmented with the standardized representations. At the prediction level, two equal-weight fusions of saved test predictions were formed: all eight learned models, and an exploratory best linear/tree/deep trio. The eight-model fusion involves no selection and is therefore the primary integration result. Finally, text-only and category-only ridge ablations complete the eighteen predictors. The two ablations serve as feature diagnostics and are excluded from model-level comparisons.

Two further predictors, trained post hoc, form a pretrained-embedding control. Each description was represented by the mean of its pretrained fastText word vectors (wiki-news, 300 dimensions) [66,67], concatenated with the category encoding. These vectors are external constants, so no representation is learned from the corpus. Ridge and XGBoost heads were then trained and permuted under the identical frozen protocol. The control tests whether the deep networks lose because their representations lack pretraining or because dense representations underperform at this data scale. Standardization statistics again derive from the fit partition only.

### 2.4. Evaluation and Diagnostics

All models were scored on the held-out test partition of every regime and seed. The metrics are the coefficient of determination ($R^{2}$), root mean square error, mean absolute error, and Spearman rank correlation (ρ) on the log(1+FRAP) scale. The results are reported as mean ± standard deviation (SD) over the five seeds. For every trained benchmark model and split, a permutation control was obtained by shuffling training-pool targets and retraining; test labels remain intact. Any control exceeding chance would expose leakage in the pipeline. Three protocol details are stated for transparency. First, the boosted and deep models were trained on the fit partition, with the validation split reserved for early stopping. In contrast, ridge, *k*-NN, and the random forest were refitted on the full training pool. Cross-family comparisons therefore differ by approximately 15% of effective training data in favor of the latter group. A fit-only control, reported in Section 3.3, retrained ridge, *k*-NN, and the forest on the fit partition alone to remove this asymmetry. Second, for the two-stage hybrids, the permutation control covered the downstream learner. The representation-producing networks carried their own permutation runs, and a fully end-to-end permutation was not performed. Third, the validation split served a double duty in the hybrids. It was used once for early stopping of the representation networks and once for selection in the downstream learner. This double use is legitimate but noted for completeness. The diagnostics comprise the leakage inflation $\Delta R^{2}$ (random minus grouped), the memorization decomposition of the *k*-NN advantage, and the feature ablations.

## 3. Results

This section presents the benchmark (Section 3.1), the leakage mechanism (Section 3.2), representation versus architecture (Section 3.3), and the integration strategies (Section 3.4).

### 3.1. Benchmark Under Both Regimes

**Figure 4:**
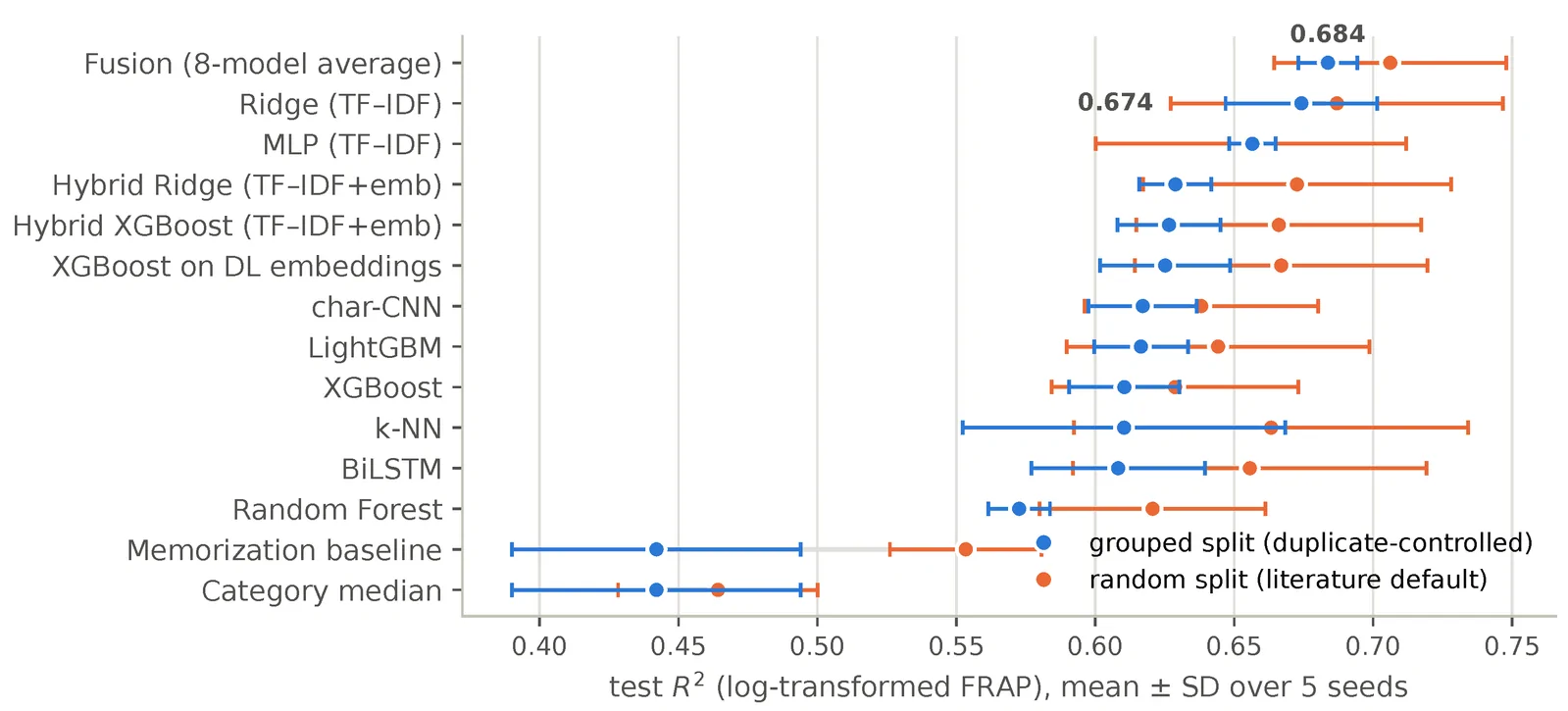
Test $R^{2}$ under the grouped (blue) and random (orange) regimes, mean ± SD over the same five seeds.

Table 1 reports the complete benchmark, and Figure 4 visualizes it. Under the grouped regime, the best individual model was ridge regression on TF–IDF and category features ($R^{2}=0.674\pm 0.027$, ρ=0.828). Overall, the eight-model fusion improved on this result, with $R^{2}=0.684\pm 0.011$ (ρ=0.845). The category median alone achieved 0.442, so the description contributes information well beyond the category. The text-only and category-only ablations reached 0.597 and 0.503, respectively, showing that the two inputs are complementary. All permutation controls remained at or below chance level (all per-model means ≤+0.004), supporting pipeline integrity. On the original scale, the grouped ridge model showed a median absolute error of 0.36±0.03 mmol/100 g. The mean absolute error was 9.4±1.5 mmol/100 g, and the fusion attained 0.33 and 9.2, respectively. The gap between the median and the mean reflects the heavy right tail of the FRAP distribution. Errors on concentrated products dominate the mean, which motivated modeling on the log(1+x) scale.

A further control examined the random forest, the only learned model trained without any validation feedback. A post hoc grid therefore varied features per split and minimum leaf size (nine configurations per regime and seed). Validation selection chose 3000 features per split in every case. Tuning raised the grouped forest from 0.573±0.011 to 0.656±0.021, still below ridge regression on all five paired seeds (−0.018±0.008). It also reduced the forest’s leakage inflation from +0.048 to +0.022. Every fixed-configuration refit reproduced the frozen benchmark to machine precision. The grid is a selection diagnostic and carries no permutation runs. The tuned forest therefore narrows the gap but does not change the ranking; Table 1 reports the fixed configuration used throughout.

Performance is heterogeneous across categories and strata. Over the pooled grouped test sets, per-category $R^{2}$ ranges from 0.69 (desserts and cakes) to negative values. Ten of the 23 categories have negative $R^{2}$; the largest deficits occur for fish and seafood, mixed food entrees, and meat and meat products. Negative values arise because the reference there is the category’s own test mean, a stringent within-category baseline. Median absolute errors remain below 0.45 mmol/100 g in 18 of the 23 categories with at least 20 test records. The largest errors concentrate where FRAP itself is high: spices and herbs (5.1), vitamin and dietary supplements (18.3), and herbal/traditional plant medicine (22.4). The high-FRAP stratum (≥10 mmol/100 g, 12.9% of test records) is the hardest: rank correlation falls to 0.27, and 78% of these products are underpredicted. The model therefore compresses the upper range, and concentrated products are systematically underestimated.

### 3.2. Leakage Inflation and Its Mechanism

**Figure 5:**
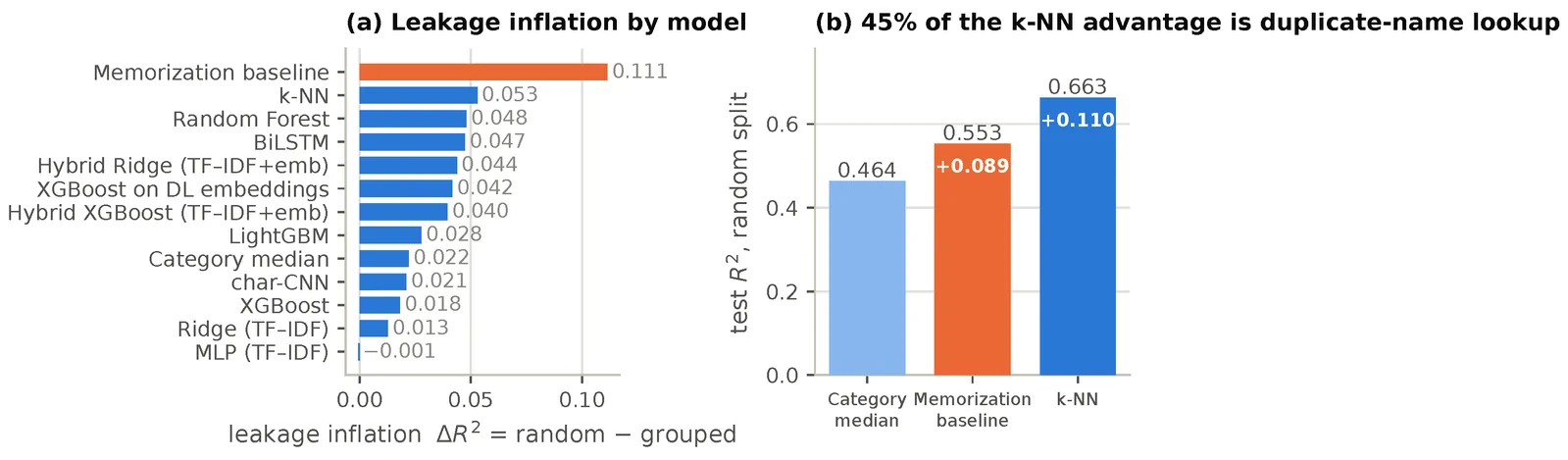
The leakage mechanism: (**a**) inflation $\Delta R^{2}$ by model; (**b**) decomposition of the *k*-NN advantage under the random regime.

Switching from the grouped to the random regime inflated the apparent performance of every main-benchmark learned model except the MLP. The inflation $\Delta R^{2}$ ranged from −0.001 to +0.053 (Figure 5a). Notably, the inflation follows a mechanistic gradient. It is largest for memorizing models (*k*-NN +0.053, random forest +0.048, BiLSTM +0.047) and smallest for ridge regression and the MLP (+0.013 and −0.001). The memorization baseline provides the ceiling. With no features and no learning, duplicate lookup alone gained +0.111.

Figure 5b decomposes the mechanism. Under the random regime, *k*-NN exceeded the category median by +0.199 in $R^{2}$ (0.663 versus 0.464). The memorization baseline reproduced +0.089 of that margin, or 45%, by retrieving identically named training records alone. Hence, nearly half of the apparent nearest-neighbor advantage with a random split reflects duplicate-name lookup. The inflation is nevertheless bounded in this dataset. Name duplicates are different brands or lots with genuinely different measured values. A resource with exact duplicated measurements would suffer correspondingly larger distortion.

### 3.3. Representation Versus Architecture

In duplicate-controlled evaluation, neither deep network matched the engineered representation. The char-CNN and BiLSTM achieved 0.617±0.019 and 0.608±0.031, respectively. Each fell below ridge regression on all five paired seeds (Table 2). The mean paired differences were −0.057 and −0.066, respectively. The most informative comparison is the MLP, which was trained on the same TF–IDF features as ridge. It came within −0.018±0.034 of ridge regression, a difference not resolvable at five seeds (p=0.38, exact sign test). In contrast, the networks that learned their representation from approximately 2100 short product descriptions trailed by at least three times that margin. These results are consistent with a representation-driven deficit at this sample size. The MLP also differs from the deep networks in depth, optimization, and regularization, so architecture and training procedure are not fully excluded.

A fit-only control removes the training-data asymmetry noted in Section 2.4. Retrained on the fit partition alone, ridge regression reached 0.678±0.021, within noise of its pool-refit value. It still exceeded the char-CNN and BiLSTM on all five paired seeds (+0.061 and +0.070). The *k*-NN and forest counterparts moved by less than 0.006. The reported ranking is therefore not an artifact of the 15% training-data difference.

The pretrained-embedding control provides a complementary test. Mean-pooled fastText vectors with the XGBoost head reached 0.628±0.033, on par with the corpus-trained deep representations with the same head (0.625±0.023). The ridge head on the same vectors reached only 0.516±0.027. Both variants remained below ridge regression on all five paired seeds (Table 2). External pretraining therefore does not close the representation gap at this data scale. The deficit follows the dense generic representations, whether pretrained or corpus-trained, which further supports the representation-centered reading.

Ridge regression also offers direct interpretability. Table 3 lists the word features with the largest mean coefficients across the five grouped seeds. The positive terms are chemically coherent: clove, amla, walnut, and dried forms mark the table’s most potent products. The negative terms include fresh preparations, oils, and dosage tokens such as mg and mcg. Brand fragments also appear, showing that the model partly exploits brand vocabulary as a proxy signal. Opposing signs among related features (fresh versus leaves fresh) and negative weights for individual spices reflect that coefficients act relative to the category encoding and co-occurring terms.

### 3.4. ML–DL Integration

**Figure 6:**
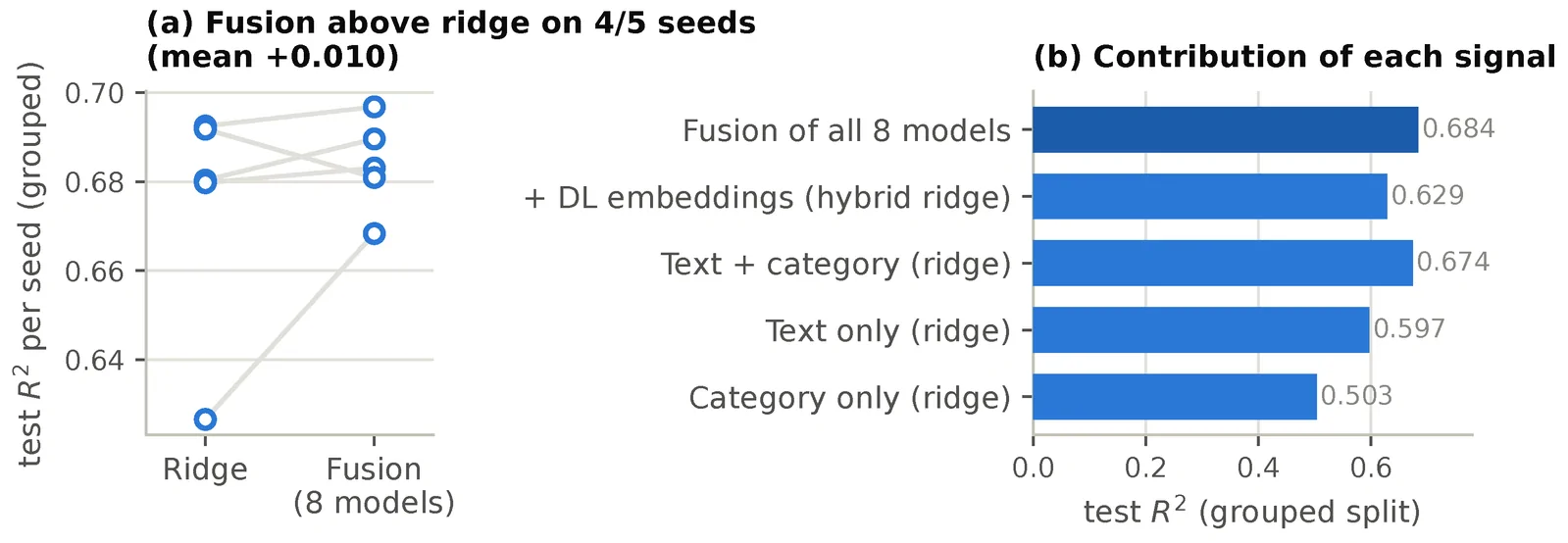
ML–DL integration: (**a**) paired per seed comparison of ridge and the eight-model fusion; (**b**) contribution of each signal.

The representation-level hybrids were informative but not competitive. XGBoost trained on the deep representations alone reached 0.625±0.023. This exceeds XGBoost on raw TF–IDF features (0.610±0.020) and indicates that the networks learned transferable structure. However, injecting the representations into the best individual model was counterproductive. The hybrid ridge fell from 0.674 to 0.629±0.013. In contrast, prediction-level integration fared better. The equal-weight fusion of all eight learned models was the only selection-free predictor to surpass ridge, doing so on four of five seeds (mean +0.010; Figure 6a). With five seeds, this mean advantage is not statistically significant. The fusion’s clearer benefit is stability. It also reduced the seed-to-seed SD from 0.027 to 0.011, a factor of 2.6. The deep networks therefore contributed diversity that the ensemble converted into stability and a small mean gain, despite being individually inferior. Figure 6b summarizes the ablation ladder. Performance rises from category-only (0.503) through text-only (0.597) and the full feature set (0.674) to the fusion (0.684).

In summary, four findings emerge. Random splitting inflates apparent performance. Duplicate lookup explains much of the inflation. Engineered features outperform learned representations at this data scale. Selection-free prediction-level fusion is the integration strategy that gives the best observed grouped-regime performance on this benchmark.

## 4. Discussion

The discussion covers the dominance of protocol and representation, the implications for users of the table, and the limitations.

### 4.1. Protocol and Representation Outweigh Architecture on This Benchmark

Taken together, the results challenge the conventional reading of ML benchmarks in food science. On this dataset, the evaluation protocol and feature representation influenced the reported outcome more than model architecture. The regime spread reached +0.053 $R^{2}$ for a standard model class, and a learning-free lookup accounted for 45% of the apparent nearest-neighbor advantage. Meanwhile, most of the 0.10 $R^{2}$ span across main-benchmark learned models reflects the untuned forest, which validation tuning lifts to 0.656. The two deep architectures differ by only 0.009. These observations extend the warnings of Kapoor and Narayanan [55] and the food-chemistry case study of Hussein et al. [57]. To the author’s knowledge, they provide the first decomposition attributing the inflation on such a resource to its exact mechanism.

The leading performance of ridge regression on TF–IDF features agrees with prior findings. Learned representations need far more data than approximately 2100 short training texts. The MLP control points primarily to the representation. The pretrained-embedding control extends this: even externally pretrained static vectors did not close the gap. Conversely, the eight-model fusion shows that deep models need not be individually superior to add value. Their errors are decorrelated enough that equal-weight averaging improved the mean slightly and the stability robustly. Practitioners who need a dependable estimate should prefer the fusion; where simplicity matters, ridge on TF–IDF features is a strong, interpretable default.

### 4.2. Implications for the Use of the Antioxidant Food Table

The results carry a practical implication for the dietary TAC literature, in which questionnaire items are matched to the table manually [39,42,54]. Matching questionnaire items to database entries is a retrieval task, which the present regression experiments do not directly validate. The benchmark nevertheless quantifies the text signal such a matcher could exploit, and the portion that stems from trivially matching identical names. A dedicated entity-linking evaluation remains future work. Studies that validate an automated matcher on randomly held-out foods will overstate its accuracy on genuinely novel items. The grouped protocol introduced here provides the appropriate estimate. A broader recommendation follows from the 39.4% duplicate share. Any predictive claim built upon the table, or upon similarly structured composition databases, should be accompanied by a grouped evaluation. Ideally, it should include a memorization baseline, whose grouped-regime equivalence to the category median also serves as a correctness check. The protocol is deliberately simple to adopt for researchers outside the ML field. It requires partitioning by product name rather than by record, together with a comparison against the duplicate-lookup and category-median baselines. The released splits and code implement both steps, so the procedure requires no specialized ML expertise.

### 4.3. Limitations

This study has limitations. First, the target variable derives from a single assay (FRAP) applied once per product. Assay-to-assay variability and analytical uncertainty are therefore not represented. FRAP itself measures reducing capacity rather than any physiological effect [1,38]. Accordingly, the models predict a compositional quality indicator, not a health outcome. The evaluation protocol, however, is not tied to the assay. Leakage arises from the data structure, in which one food recurs across brands, lots, and origins, not from the assay chemistry. Composition databases built on the 2,2-diphenyl-1-picrylhydrazyl (DPPH), 2,2′-azino-bis(3-ethylbenzothiazoline-6-sulfonic acid) (ABTS), or ORAC assays are assembled from the same market-sampling practice [1]. Comparable duplication can therefore be expected, whether as repeated brands and lots or as multiple rows per food, as in the ORAC study cited above. Grouping by food identity, the permutation controls, and the memorization baseline transfer to both layouts unchanged. Second, the inputs are limited to the product description and category. Compositional predictors such as the polyphenol profiles in Phenol-Explorer [27,28] could not be joined at scale. That database describes raw commodities, whereas the Antioxidant Food Table is dominated by branded, processed products. This mismatch marks an open-data gap. Third, the deep models were deliberately compact and CPU-trainable. A lightweight pretrained-embedding control showed that static word vectors do not close the representation gap. Whether large contextual language models would perform better remains untested. Accordingly, the conclusions cover the evaluated character-level networks and static embeddings at this data scale, not deep text representations in general. Fourth, three protocol asymmetries were stated in Section 2.4. These are the fit-versus-pool training difference, the downstream-only permutation of the hybrids, and the double use of the validation split. They temper fine-grained cross-family comparisons, although none affects the headline contrasts between regimes. Finally, the conclusions are drawn from a single, albeit uniquely large, food antioxidant resource. No external-dataset validation was performed, so cross-resource generalization remains unquantified. Replication on other composition databases is desirable; the released code makes it straightforward.

## 5. Conclusions

This study converted the most widely used food antioxidant capacity resource into an open benchmark that measures, rather than assumes, the effect of evaluation protocol. The conclusions of this study can be given as follows:

(i) The Antioxidant Food Table was released in machine-readable form for the first time, covering 3135 of 3139 records (99.9%). An independent value-level census confirms that the extraction is faithful to the printed supplement in 23 of 24 categories. The single shortfall is one record the extractor did not recover. Every difference from the article’s summary table traces to the source.
(ii) The literature-standard random split systematically inflates performance, because 39.4% of records share a normalized product name. The inflation tracks model memorization capacity, reaching +0.053 $R^{2}$ for *k*-NN. A learning-free memorization baseline reproduces 45% of the apparent nearest-neighbor advantage.
(iii) In duplicate-controlled evaluation, ridge regression on TF–IDF features ($R^{2}=0.674\pm 0.027$) outperformed the character-level CNN and BiLSTM networks on every paired seed. The representation-matched MLP indicates that the deficit is primarily representational rather than architectural. A pretrained-embedding control further supports this reading: externally pretrained static vectors did not close the gap either.
(iv) ML–DL integration proved effective at the prediction level. The selection-free fusion of all eight learned models achieved the best observed grouped-regime performance ($R^{2}=0.684\pm 0.011$; Spearman ρ=0.845). It surpassed ridge regression on four of five seeds while reducing seed-to-seed variability by a factor of 2.6. The mean gain is small; the robust benefit is the reduced variability.

Future work will extend the benchmark with compositional inputs via a processed-food bridge to polyphenol composition databases. Pretrained language models will then be evaluated under the same frozen protocol, to test whether contextual pretraining succeeds where static pretrained vectors did not. Extending the benchmark to resources measured with other antioxidant assays is a further priority. The protocol and memorization baseline will also be applied to further composition resources, for which the released materials provide a complete template.

## Figures and Tables

**Table 1:** Test $R^{2}$ (mean ± SD over the same five seeds) under both regimes and the leakage inflation $\Delta R^{2}$. Bold marks the highest grouped-regime mean.

Model	Grouped $R^{2}$	Random $R^{2}$	$\Delta R^{2}$
*Baselines*			
Global median	−0.216±0.025	−0.202±0.015	+0.014
Category median	0.442±0.052	0.464±0.036	+0.022
Memorization (duplicate lookup)	0.442±0.052	0.553±0.027	+0.111
*Classical ML*			
Random forest	0.573±0.011	0.621±0.041	+0.048
*k*-NN	0.610±0.058	0.663±0.071	+0.053
XGBoost	0.610±0.020	0.629±0.044	+0.018
LightGBM	0.616±0.017	0.644±0.055	+0.028
MLP (TF–IDF)	0.657±0.008	0.656±0.056	−0.001
Ridge regression (TF–IDF)	0.674±0.027	0.687±0.060	+0.013
*DL*			
BiLSTM	0.608±0.031	0.656±0.064	+0.047
char-CNN	0.617±0.019	0.638±0.042	+0.021
*ML–DL integration*			
XGBoost on representations	0.625±0.023	0.667±0.053	+0.042
Hybrid XGBoost (TF–IDF + repr.)	0.627±0.019	0.666±0.051	+0.040
Hybrid ridge (TF–IDF + repr.)	0.629±0.013	0.673±0.055	+0.044
Fusion of best linear/tree/deep	0.679±0.013	0.694±0.046	+0.015
Fusion of all eight models	0.684±0.011	0.706±0.042	+0.022
*Feature ablations (ridge)*			
Text only	0.597±0.026	0.634±0.055	+0.037
Category only	0.503±0.065	0.494±0.032	−0.009

**Table 2:** Paired per seed comparison against ridge regression under the grouped regime.

Model	Mean Difference ± SD	Seeds Above Ridge
Fusion of all eight models	+0.010±0.019	4/5
Fusion of best linear/tree/deep	+0.005±0.017	3/5
MLP (TF–IDF)	−0.018±0.034	1/5
Hybrid ridge (TF–IDF + repr.)	−0.045±0.030	0/5
fastText + XGBoost (pretrained)	−0.047±0.031	0/5
Hybrid XGBoost (TF–IDF + repr.)	−0.048±0.034	0/5
XGBoost on representations	−0.049±0.038	0/5
char-CNN	−0.057±0.033	0/5
LightGBM	−0.058±0.013	0/5
*k*-NN	−0.064±0.031	0/5
BiLSTM	−0.066±0.007	0/5
fastText + ridge (pretrained)	−0.158±0.026	0/5

**Table 3:** Word features with the largest mean ridge coefficients over the five grouped seeds.

Positive		Negative	
**Feature**	**Coefficient**	**Feature**	**Coefficient**
tegreen	+1.36	mg	−1.08
clove	+1.27	fresh	−0.78
walnuts with	+1.24	coriander	−0.76
amla	+1.04	mcg	−0.75
antioxidant	+1.02	oil	−0.73
clove whole	+0.89	sano	−0.73
cvs	+0.87	coneflower	−0.67
leaves fresh	+0.84	ginseng	−0.66
dried	+0.82	cardamom	−0.65
rose	+0.78	nature	−0.62

## Data Availability

The primary antioxidant capacity data were obtained from the work of Carlsen et al. [37] and are freely available at https://doi.org/10.1186/1475-2891-9-3 (accessed on 10 August 2026). The machine-readable dataset derived in this study is permanently archived on Zenodo (https://doi.org/10.5281/zenodo.21896513) under the CC-BY license. All source code, the frozen split definitions, model settings, random seeds, per-run prediction files, and learned representations are freely available at https://github.com/eco160/afd-leakage-aware-benchmark (accessed on 12 August 2026). A pinned environment (Python 3.12.3; scikit-learn 1.2.2, XGBoost 3.2.0, LightGBM 4.6.0, PyTorch 2.6.0, NumPy 1.26.4, SciPy 1.17.1, pandas 2.3.3) enables reproduction of every table and figure.

## Abbreviations

The following abbreviations are used in this manuscript:

ABTS	2,2′-azino-bis(3-ethylbenzothiazoline-6-sulfonic acid)
BiLSTM	Bidirectional long short-term memory
CC-BY	Creative Commons Attribution
CNN	Convolutional neural network
CPU	Central processing unit
DL	Deep learning
DPPH	2,2-diphenyl-1-picrylhydrazyl
FRAP	Ferric reducing ability of plasma
*k*-NN	*k*-nearest neighbors
ML	Machine learning
MLP	Multilayer perceptron
ORAC	Oxygen radical absorbance capacity
SD	Standard deviation
TAC	Total antioxidant capacity
TF–IDF	Term frequency–inverse document frequency
